# The effects of an editor serving as one of the reviewers during the peer-review process

**DOI:** 10.12688/f1000research.8452.2

**Published:** 2016-10-18

**Authors:** Marco Giordan, Attila Csikasz-Nagy, Andrew M. Collings, Federico Vaggi

**Affiliations:** 1 Fondazione Edmund Mach, San Michele, Italy; 2 King's College London, London, UK; 3 eLife Sciences Publications Ltd, Cambridge, UK; 4 Faculty of Information Technology and Bionics, Peter Pazmany Catholic University, Budapest, H-1083, Hungary

**Keywords:** peer review, decision times, eLife

## Abstract

**Background**

Publishing in scientific journals is one of the most important ways in which scientists disseminate research to their peers and to the wider public. Pre-publication peer review underpins this process, but peer review is subject to various criticisms and is under pressure from growth in the number of scientific publications.

**Methods**

Here we examine an element of the editorial process at *eLife*, in which the Reviewing Editor usually serves as one of the referees, to see what effect this has on decision times, decision type, and the number of citations. We analysed a dataset of 8,905 research submissions to *eLife* since June 2012, of which 2,747 were sent for peer review. This subset of 2747 papers was then analysed in detail.

**Results **

The Reviewing Editor serving as one of the peer reviewers results in faster decision times on average, with the time to final decision ten days faster for accepted submissions (n=1,405) and five days faster for papers that were rejected after peer review (n=1,099). Moreover, editors acting as reviewers had no effect on whether submissions were accepted or rejected, and a very small (but significant) effect on citation rates.

**Conclusions**

An important aspect of *eLife*’s peer-review process is shown to be effective, given that decision times are faster when the Reviewing Editor serves as a reviewer. Other journals hoping to improve decision times could consider adopting a similar approach.

## Background

Although pre-publication peer review has been strongly criticised – for its inefficiencies, lack of speed, and potential for bias (for example, see 1 and 2) – it remains the gold standard for the assessment and publication of research ^3^. *eLife* was launched to “improve [...] the peer-review process” ^4^ in the life and biomedical sciences, and one of the journal’s founding principles is that “decisions about the fate of submitted papers should be fair, constructive, and provided in a timely manner” ^5^. However, peer review is under pressure from the growth in the number of scientific publications, which increased by 8–9% annually from the 1940s to 2012 ^6^, and growth in submissions to *eLife* would inevitably challenge the capacity of their editors and procedures.

*eLife* ( https://elifesciences.org/) was launched in 2012 to publish highly influential research across the life sciences and biomedicine; research articles in *eLife* are published within 15 broad subject areas, including cell biology and neuroscience (with the most publications), through to ecology and epidemology/global health (with fewer publications; https://elifesciences.org/search).

*eLife’*s editorial process has been described before ^7, 8^. In brief, each new submission is assessed by a Senior Editor, usually in consultation with one or more members of the Board of Reviewing Editors, to identify whether it is appropriate for in-depth peer review. Traditionally, editors recruit peer reviewers and, based on their input, make a decision about the fate of a paper. Once a submission is sent for in-depth peer review, however, the Reviewing Editor at *eLife* has extra responsibility.

First, the Reviewing Editor is expected to serve as one of the peer reviewers. Once the full submission has been received, it is assigned by staff to the Reviewing Editor who agreed to handle it, both as the handling editor, and as one of the reviewers, unless the Reviewing Editor actively decides against serving as a referee. A common reason for not serving as one of the referees is workload: for example, a Reviewing Editor already handling two papers as an editor and a reviewer may be less likely to take on a third, unless they can take the third one without providing a review. Another common reason for not serving as one of the referees is when the paper is outside of the Reviewing Editor’s immediate area of expertise: however, *eLife* editors are still encouraged to serve as a reviewer in these circumstances as a review from this perspective can be informative in helping to assess a paper’s broad appeal. We cannot rule out the possibility that some Reviewing Editors self select the most interesting submissions to provide a review themselves, but the journal takes various steps to encourage the practice of providing a review wherever possible: for example, by tracking the trend on a monthly basis, by explaining this expectation when a Reviewing Editor first joins, and by asking for a justification when Reviewing Editors decides against providing a review of his or her own.

Second, once the reviews have been submitted independently, the Reviewing Editor should engage in discussions with the other reviewers to reach a decision they can all agree with. Third, when asking for revisions, the Reviewing Editor should synthesise the separate reviews into a single set of revision requirements. Fourth, wherever possible, the Reviewing Editor is expected to make a decision on the revised submission without re-review. At other journals, the Reviewing Editor may instead be known as an Academic Editor or Associate Editor.

Since editors have extra responsibility in *eLife*’s peer-review process, here we focus our analysis on the effect of the Reviewing Editor serving as one of the peer reviewers, and we examine three outcomes: 1) the effect on decision times; 2) the effect on the decision type (accept, reject or revise); and 3) the citation rate of published papers. The results of the analysis are broken down by the round of revision and the overall fate of the submission. We do not consider the effect of the discussion between the reviewers or the effect of whether the Reviewing Editor synthesizes the reviews or not.

## Methods

We analysed a dataset containing information about 9,589 papers submitted to *eLife* since June 2012 in an anonymised format. The dataset contained the date each paper was first submitted, and, if it was sent for peer review, the dates and decisions taken at each step in the peer-review process. Information about authors had been removed, and the identity of reviewers and editors was obfuscated to preserve confidentiality. This dataset was obtained in collaboration with the editorial staff at *eLife*, who contributed to and collaborated on this manuscript.

As a pre-processing step, we removed papers that had been voluntarily withdrawn, or where the authors appealed a decision, as well as papers where the records were corrupted or otherwise unavailable. After clean up, our dataset consisted of a total of 8,905 submissions, of which 2747 were sent for peer review. For the rest of the paper, we focus our analysis on this subset of 2747 papers, of which 1,405 had been accepted, 1,099 had been rejected, and the rest were still under consideration. The article types included are Research Articles (MS type 1), Short Reports (MS type 14), Tools and Resources (MS type 19), and Research Advances (MS type 15). Registered Reports are subject to a slightly different review process and have not been included.

Before discussing the results, we introduce a few definitions: the “ *eLife* Decision Time” is the amount of time taken by *eLife* from the version of the submission being received until a decision has been reached for a particular round of review. The “Author Time” is the amount of time taken by the authors to revise their article for that round of revision. The “Total Time” is the time from first submission to acceptance, or amount of time taken for *eLife* to accept a paper from the moment it was first received for consideration. By definition, the “Total Time” is equal to the sum of the “ *eLife* Decision Time” and the “Author Time” across all rounds, including the initial submission step. “Revision Number” indicates the round of revision. We distinguish between Reviewing Editors who served as one of the reviewers during the first round of review and Reviewing Editors who did not serve as one of the reviewers (i.e., did not personally try to critically evaluate the scientific content of the article) with the “Editor_As_Reviewer” variable (True or False).

We illustrate the variables with a real example taken from the dataset ( Table 1).

**Table 1.** An example from the dataset.

MS Type	Revision Number	Received Date	Decision Date	*eLife* Decision Time	Total Time	Author Time	Editor_As_ Reviewer
5	1	2012-06-20	2012-06-21	1	N/A	N/A	
1	1	2012-06-27	2012-07-25	28	N/A	6	True
	2	2012-09-05	2012-09-05	0	77	42	True

The example submission from Table 1 was received as an “initial submission” (MS TYPE 5) on 20th June 2012. One day later, the authors were encouraged to submit a “full submission” (MS TYPE 1) that would be sent for in-depth peer review. The full submission was received on 27th June 2012, when the Reviewing Editor was assigned and reviewers were contacted. In this example, the Reviewing Editor also served as one of the reviewers (indicated by the “Editor_As_Reviewer” variable).

On 25th July (28 days later), the Reviewing Editor sent out a decision asking for revisions to the authors, who submitted their revised manuscript on 5th September. The paper was accepted on the same day that it was resubmitted. In this case, the total *eLife* Decision Time was 29 days (including the pre-review stage), the Author Time was 48 days, and the Total Time ( *eLife* Decision Time plus Author Time) was 77 days. Total Time refers only to the total time across all rounds and revisions for each paper - and therefore does not vary across rounds. Since we are focusing on the role of the editors in the peer review process, in the rest of the paper we will ignore the time spent in the pre-review stage.

All of the statistical analyses were performed using R and Python. On the Python side, we used statsmodels, scipy, numpy, and pandas for the data manipulation and analysis. To plot the results we used bokeh, matplotlib, and seaborn. Details of all the analysis, together with code to reproduce all image and tables in the paper are available on the companion repository of this paper here: https://github.com/FedericoV/eLife_Editorial_Process.

To obtain the citation numbers, we used a BeautifulSoup to scrape the *eLife* website, which provides detailed information about citations for each published paper.

## Results and discussion

**Figure 1.**
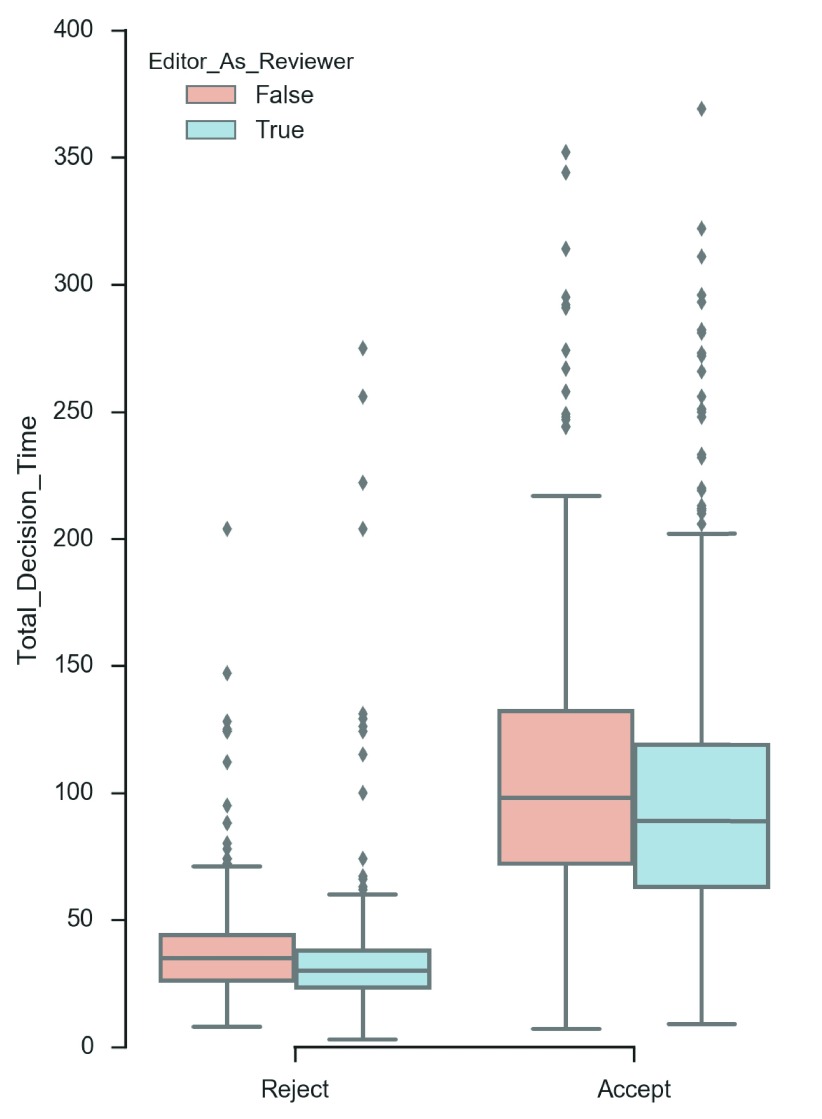
Decision times are faster when the Reviewing Editor serves as one of the reviewers. We compare the total time from submission to acceptance and submission to rejection after peer review. Light blue indicates papers submissions where the Reviewing Editor served as one of the peer reviewers, while orange indicates submissions where the Reviewing Editor did not serve as one of the reviewers (i.e., the editors had more of a supervisory role).

First, we examined the effect of the Reviewing Editors serving as one of the reviewers on the time from submission to acceptance or from submission to rejection after peer review (Total Time). When the Reviewing Editor served as a reviewer (Editor_As_Reviewer = True), the total processing time was 10 days faster in the case of accepted papers and more than 5 days faster in the case of papers rejected after peer review ( Figure 1). Both differences are statistically significant (see Table 2 for details). Intuitively, regardless of the role of the Reviewing Editor, rejection decisions are typically much faster than acceptance decisions, as they go through fewer rounds of revision, and are not usually subject to revisions from the authors.

**Table 2.** Effect of a Reviewing Editor serving as a reviewer (Editor_As_Reviewer) on *eLife* Decision Time and Author Time.

		Counts		*eLife* Decision Time			Author Time		
	Editor_As_Reviewer	False	True	False	True	M-W	False	True	M-W
Decision_Type	Revision Number								
**Accept Full** **Submission**	**0**	5.000	12.000	21.600	23.333	1.000	4.000	4.833	0.915
	**1**	440.000	650.000	8.802	6.949	0.006	52.209	51.168	0.402
	**2**	115.000	164.000	4.339	3.238	0.175	16.487	14.652	0.402
	**3**	6.000	11.000	3.667	2.455	0.747	6.833	9.909	0.811
**Reject Full** **Submission**	**0**	461.000	616.000	36.104	30.981	0.000	6.182	6.430	0.267
	**1**	10.000	10.000	22.200	31.600	0.148	64.900	101.800	0.402
	**2**	1.000	N/A	17.000	N/A	0.000	60.000	N/A	0.000
	**3**	N/A	N/A	N/A	N/A	N/A	N/A	N/A	N/A
**Revise Full** **Submission**	**0**	705.000	946.000	36.018	31.053	0.000	5.786	5.744	0.402
	**1**	129.000	182.000	19.651	15.747	0.006	66.930	64.110	0.915
	**2**	6.000	12.000	7.833	7.167	0.747	21.333	35.250	0.730
	**3**	N/A	N/A	N/A	N/A	N/A	N/A	N/A	N/A

One possible reason why submissions reviewed by the Reviewing Editor have a faster turnaround is because fewer people are involved (e.g., the Reviewing Editor in addition to two external reviewers, rather than the Reviewing Editor recruiting three external reviewers), and review times are limited by the slowest person. To test this, we built a linear model to predict the total review time as a function of editor type (whether the Reviewing Editor served as a reviewer or not), decision (accept or reject), and the number of unique reviewers across all rounds (see Table S1). Indeed, the total review time did increase with each reviewer (7.4 extra days per reviewer, p < 0.001) and the effect of a Reviewing Editor serving as one of the reviewers remained significant (–9.3 days when a Reviewing Editor served as one of the reviewers, p < 0.0001). Additionally, another possibility is that the papers with more reviewers were more technically challenging, and so required more review time to fully examine all the complexity.

**Figure 2.**
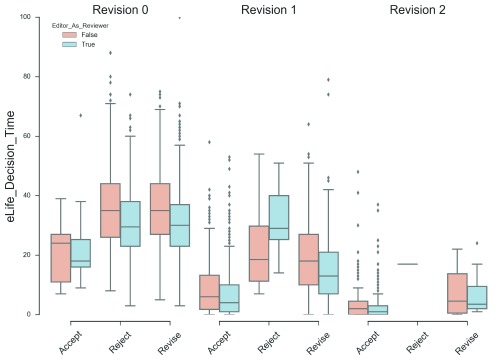
Decision times are faster when the Reviewing Editor serves as one of the reviewers across different rounds of review. Boxplots showing decision times for different rounds of review, depending on decision type and whether the Reviewing Editor served as one of the reviewers (light blue) or not (orange). Full data available in Table 2.

Next, we examined this effect across all rounds of review (rounds 0, 1, 2) and decision types (accept, reject and revise). The results are shown in Figure 2 and summarised in Table 2. Again, we see that processing times are consistently faster across almost every round, when the editors serves as one of the peer reviewers, except in the cases where the sample size was very small.

Interestingly, when the Reviewing Editor serves as one of the peer reviewers, the *eLife* Decision Time is reduced, but the time spent on revisions (Author Time) does not change. This suggests that the actual review process is more efficient when the Reviewing Editor serves as a reviewer, but the extent of revisions being requested from the authors remains constant.

We next examined the chances of a paper being accepted, rejected or revised when a Reviewing Editor served as one of the reviewers. We found no significant difference when examining the decision type on a round-by-round basis ( Table 3) (chi-squared test, p = 0.33).

**Table 3.** Effect of a Reviewing Editor serving as one of the reviewers on editorial decisions.

Editor_As_Reviewer	False	True	Totals
**Accept Full Submission**	566	837	1403
**Reject Full Submission**	472	626	1098
**Revise Full Submission**	840	1140	1980
**Totals**	1878	2603	4481

To test whether *eLife*’s acceptance rate changed over time, we built a logit model including as a predictive variable the number of days since *eLife* began accepting papers and whether the Reviewing Editor served as one of the reviewers. In this model, we test whether the dependent variable (the probability that a paper is published by *eLife*) is affected by the number of referees reviewing a paper ( **Unique_Reviewers**), whether the Reviewing Editor was also serving as a reviewer ( **Editor_as_Reviewer**), and the number of days since *eLife* began accepting papers ( **Publication_Since_Start**).

The only significant variable in our analysis was the number of days since publication ( **Publication_Since_Start)**, which had a very small (-0.003) but significant effect (p < 0.02) (see **Table S2**). That is to say that the chances of a paper submitted to *eLife* being accepted have declined over time. It’s important however to highlight that we cannot say whether this trend reflects changes in *eLife’*s acceptance criteria without assuming that the average quality of papers *eLife* has remained constant. As the volume of papers processed by *eLife* has greatly increased over three years, this is a very difficult factor to independently verify - as such, while we report all the analysis and include the full dataset as well as the scripts to reproduce them, we suggest caution when interpreting the results.

**Figure 3.**
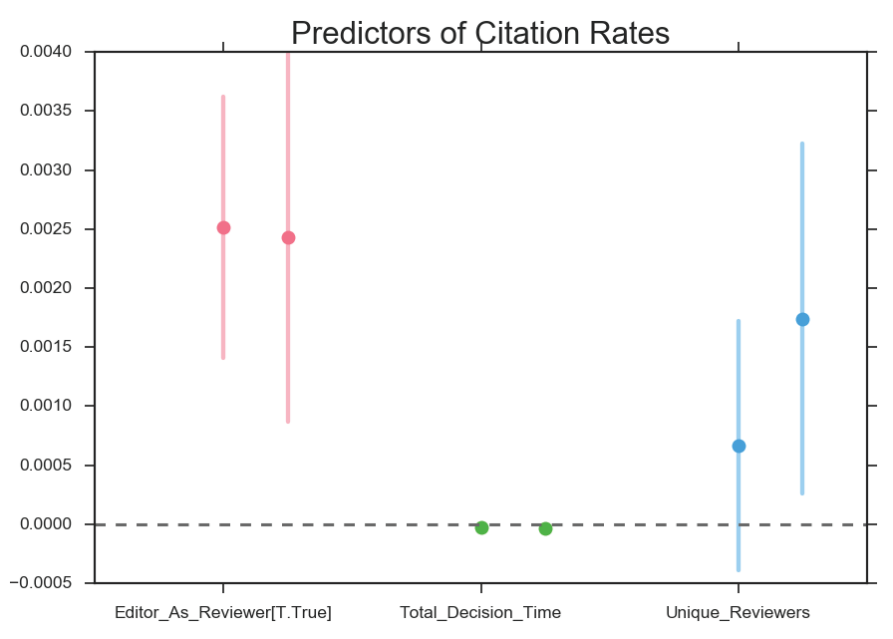
Effect of different factors on citation rates. We compare the effect of different parameters on the log1p citation rate (log(1 + (number of citations/days since paper was published)). The values of the coefficient on the left reflect the citations numbers (as indexed by Scopus) on 29th February, 2016. We now repeat this analysis using more recent citation numbers obtained 11th July 2016 which are the values on the right).

The final outcome we examined was the number of citations (as tracked by Scopus) received by papers published by *eLife*. Papers accumulate citations over time, and, as such, papers published earlier tend to have more citations ( Figure 3).

We examined this effect using a generalised linear model. As variables, we considered whether the Reviewing Editor served as a reviewer (Editor_As_Reviewer), the total amount of time taken to review the paper (Total_Decision_Time) as well as the number of reviewers examining the paper (Unique_Reviewers).

We take advantage of the ability to upload new manuscript versions to repeat our analysis using updated citation data. We report the coefficients calculated using the original citation dataset, obtained on 29th February 2016, as well as using more recent citation data obtained on 11th July. The coefficients estimated in both instances have overlapping confidence intervals, but, if we only look at the latest dataset, then the effect of the number of reviewers on citation is statistically significant (although barely; see Table S4) and papers with more reviewers tend to gather slightly more citations over time. The presence of a Reviewing Editor serving as a reviewer had lead to a small increase in citations using both citation datasets (see Table S4). Papers with longer total review times tended to be cited less (this effect is small but significant). We counsel caution when interpreting these results: the confidence intervals are quite large, and the effect size is small ( Figure 3, red dots).

**Figure 4.**
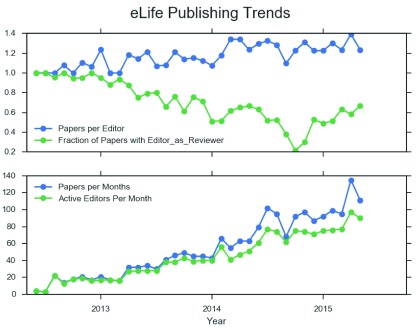
A decreasing proportion of Reviewing Editors served as one of the reviewers as submission volumes increased. The average number of papers per editor, and the number of editors willing to act as a reviewer on papers has decreased over time. The total number of papers and the number of active reviewers have both been increasing over time, although the number of active reviewers has not quite kept up with the number of papers.

One of the most noticeable effects of a Reviewing Editor serving as one of the peer reviewers at *eLife* is the faster decision times. However, serving as a Reviewing Editor and one of the reviewers for the same submission is a significant amount of work. As the volume of papers received by *eLife* has increased, the fraction of editors willing to serve as a reviewer has decreased. While in 2012 almost all editors also served as reviewers, that percentage decreased in 2013 and 2014. There are signs of a mild increase in the percentage of editors willing to serve as reviewers in 2015 ( Figure 4).

## Conclusions

Due to an increasingly competitive funding environment, scientists are under immense pressure to publish in prestigious scientific journals, yet the peer-review process remains relatively opaque at many journals. In a systematic review from 2012, the authors conclude that “Editorial peer review, although widely used, is largely untested and its effects are uncertain” ^9^. Recently, journals and conferences (e.g., 10) have launched initiatives to improve the fairness and transparency of the review process. *eLife* is one such example. Meanwhile, scientists are frustrated by the time it takes to publish their work ^11^.

We report the analysis of a dataset consisting of articles received by *eLife* since launch and examine factors that affect the duration of the peer-review process, the chances of a paper being accepted, and the number of citations that a paper receives. In our analysis, when an editor serves as one of the reviewers, the time taken during peer review is significantly decreased. Although there is additional work and responsibility for the editor, this could serve as a model for other journals that want to improve the speed of the review process.

Journals and editors should also think carefully about the optimum number of peer reviewers per paper. With each extra reviewer, we found that an extra 7.4 days are added to the review process. Editors should of course consider subject coverage and ensure that reviewers with different expertise can collectively comment on all parts of a paper, but where possible there may be advantages, certainly in terms of speed and easing the pressure on the broader reviewer pool, of using fewer reviewers per paper overall.

Insofar as the editor serving as a reviewer is concerned, we did not observe any difference in the chances of a paper being accepted or rejected, but we did notice a modest increase in the number of citations that a paper receives when an editor serves as one of the reviewers, although this effect is very small. An interesting result from our analysis is that a longer peer-review process or more referees does not lead to an increase in citations (note: using the updated citation data, there is an effect which is barely greater than zero – see Table S4, part 2), so this is another reason for journals and editors to carefully consider the impact of the number of reviewers involved, and to strive to communicate the results presented in a timely manner for others to build upon. As *eLife* is a relatively young journal, we can verify if the citations trend we observe will hold over longer periods as different papers accumulate citations.

## Data and software availability

All code for the analysis as well as the datasets: https://github.com/FedericoV/eLife_Editorial_Process

Archived version as at the time of publication: http://dx.doi.org/10.5281/zenodo.160716 ^15^

To reproduce Figure 4, we pre-processed the raw dataset that contained the identity of the editors to avoid disclosing any information about the identity of reviewers.

## Figures and Tables

**Table S1.** Linear model for Total Time. We built a linear model of the Total Time as a function of whether the Reviewing Editor served as a reviewer (Editor_As_Reviewer) (categorical variable, two levels), the final decision made on a paper (Decision_No), and the number of unique reviewers. The revision time increased with the number of reviewers, but it decreased when a Reviewing Editor served as one of the reviewers.

	coef	std err	z	P>|z|	[95.0% Conf. Int.]
**Intercept**	85.7868	4.462	19.225	0.000	77.041 94.533
**C(Editor_As_** **Reviewer)[T.True]**	-9.2265	1.701	-5.425	0.000	-12.560 -5.893
**C(Decision_Type)** **[T.Reject Full** **Submission]**	-65.3500	1.622	-40.285	0.000	-68.529 -62.171
**Unique_Reviewers**	7.1892	1.620	4.438	0.000	4.014 10.364

**Table S2.** Linear model for the chances of a paper being accepted. We used logit regression to estimate the chances of a paper being accepted as a function of whether the Reviewing Editor served as one of the reviewers (Editor_As_Reviewer), the number of unique reviewers, and the number of days between when a paper was published and the first published paper by *eLife*. The only significant variable is the days since *eLife* started accepting papers for publication (although the effect on the chances of a paper being accepted is very small).

	coef	std err	z	P>|z|	[95.0% Conf. Int.]
**Intercept**	0.6080	0.257	2.368	0.018	0.105 1.111
**C(Editor_As_Reviewer)[T.True]**	0.0822	0.087	0.946	0.344	-0.088 0.252
**Publication_Since_Start**	-0.0003	0.000	-2.393	0.017	-0.001 -6.08e-05
**Unique_Reviewers**	-0.0380	0.081	-0.468	0.640	-0.197 0.121

**Table S3.** Effect of a Reviewing Editor serving as a reviewer on the number of rounds of revision. We used a GLM with a log link function to model the number of revisions that a paper undergoes prior to a final decision as a function of whether a Reviewing Editor served as one of the reviewers (Editor_As_Reviewer), the number of unique reviewers, the decision type, and the number of days since *eLife* started accepting papers. The only variable that had a significant effect was the decision type, as papers that are rejected tend to be overwhelmingly rejected early on and thus undergo fewer rounds of revision.

	coef	std err	z	P>|z|	[95.0% Conf. Int.]
**Intercept**	0.6856	0.099	6.951	0.000	0.492 0.879
**Editor_As_Reviewer[T.True]**	-0.0134	0.033	-0.403	0.687	-0.078 0.052
**C(Decision_Type)[T.Reject** **Full Submission]**	-0.7762	0.035	-22.216	0.000	-0.845 -0.708
**Publication_Since_Start**	1.058e-05	5.33e-05	0.198	0.843	-9.4e-05 0.000
**Unique_Reviewers**	0.0382	0.031	1.230	0.219	-0.023 0.099

**Table S4.** Citation rates. Linear model for the citation rates after log1p transform. We repeated the analysis using Scopus citation information collected on two different dates nearly 6 months apart. We report the coefficients for both datasets:

February 29th 2016:					
	coef	std err	z	P>|z|	[95.0% Conf. Int.]
**Intercept**	0.0038	0.002	2.538	0.011	0.001 0.007
**Editor_As_Reviewer[T.True]**	0.0025	0.001	4.465	0.000	0.001 0.004
**Total_Decision_Time**	-2.812e-05	5.27e-06	-5.338	0.000	-3.84e-05 -1.78e-05
**Unique_Reviewers**	0.0007	0.001	1.241	0.215	-0.000 0.002
July 11th 2016:
	coef	std err	z	P>|z|	[95.0% Conf. Int.]
**Intercept**	**0.0052**	**0.002**	**2.448**	**0.014**	**0.001 0.009**
**Editor_As_Reviewer[T.True]**	**0.0024**	**0.001**	**3.059**	**0.002**	**0.001 0.004**
**Total_Decision_Time**	**-3.081e-05**	**7.42e-06**	**-4.155**	**0.000**	**-4.54e-05 -1.63e-05**
**Unique_Reviewers**	**0.0017**	**0.001**	**2.302**	**0.021**	**0.000 0.003**

The effect of the number of unique reviewers on citation rates increases slightly in the second dataset, while the other coefficients remain very similar.
