# Supplementary material for: The effects of an editor serving as one of the reviewers during the peer-review process
Source: F1000Res. 2016 Oct 18;5:683. Originally published 2016 Apr 14. [Version 2] doi: 10.12688/f1000research.8452.2 (PMC4962294; doi:10.12688/f1000research.8452.2)
Supplement: Peer review history

# Peer review: Referee response for version 2

**DOI:** 10.5256/f1000research.10481.r17081

**Published:** 2016-10-20

**Authors:** Ivan Oransky, Alison Abritis

**Affiliations:** 1 Retraction Watch, The Center For Scientific Integrity, New York, NY, USA

Thanks to the authors for responding thoughtfully to all of our suggestions and questions. We are pleased to approve this article.

We have read this submission. We believe that we have an appropriate level of expertise to confirm that it is of an acceptable scientific standard.

# Peer review: Referee response for version 1

**DOI:** 10.5256/f1000research.9100.r13397

**Published:** 2016-07-26

**Authors:** Ivan Oransky, Alison Abritis

**Affiliations:** 1 Retraction Watch, The Center For Scientific Integrity, New York, NY, USA

Thank you for the opportunity to review this manuscript. This is a well-done study, and the conclusions follow from the results. We would recommend accepting the article once all clarifications and revisions have been made, or the lack of doing so adequately justified.

 A. While brevity is generally to be admired, we would recommend a bit more detail about the statistical analyses. These are critical, but are reduced to 3 sentences and a referral to the programming language through an external link. We would suggest that the main text include the (brief) discussion of the analyses done, and rationale for them, rather than have those relegated to the external link.

 B. The interpretation of the findings seems to be attributing causal factors - an A leads to B consideration - for which the control of variables is too limited. We believe that interpreting these as associations would be more consistent with the findings.

 Consider the statement: "Journals and editors should also think carefully about the optimum number of peer reviewers per paper. With each extra reviewer, we found that an extra 7.4 days are added to the review process." Given that there appeared to be no inclusion of either article quality or complexity in the evaluation, is it not possible that issues within the article itself required the use of additional reviewers (i.e. a B leads to A perspective)? Perhaps extra reviewers with specific expertise was required, or concerns with potential problems in the manuscript led to consultations with other reviewers. It does not seem safe to assume that it was the addition of the reviewer that added extra days.

 Similarly, the study centers around the role of the editor in the reviewing process, and the discussion suggested that the involvement of the reviewing editor as a peer reviewer expedited the process. There was little discussion of other factors that could have accounted for the statistical results. For example, perhaps the reviewing editor selected articles that piqued his or her interest, or were more clearly presented. Perhaps the reviewing editor selected to review at times more convenient to his or her workload, while other reviewers did not have such an option. The reviewing editor might select to review articles perceived to be of greater or timelier value to the journal itself, which may increase the speed of the review. 

 Specific questions:

 A. According to their method section, the authors state that they began with an initial N=9,589. After purging other articles they had an N=8,905. They then isolated a total of 2,750 articles subjected to the peer review process for the study: "For the rest of the paper, we focus our analysis on this subset of 2,750 papers, of which 1,405 had been accepted, 1,099 had been rejected, and the rest [which would equal 246] were still under consideration."

 Looking at the Excel spreadsheet for citation counts, there are 1407 lines with entry numbers. For peer-reviewed papers, excel spreadsheet has 2747 (after removing duplicate entries based on the MS NO column) entries for manuscripts numbered up to 12621. The excel spreadsheet for unique reviewers has 2747 entries, with a final MS NO of 12621.

 The numbers do not appear to match, and there is no explanation for that in methods. Exactly how many manuscripts were reviewed, how many rejected and why, and how many were tracked?

 B. In the Excel spreadsheet for citations, the second column was titled "Citations," but these figures do not appear to have any relation to the Scopus citation numbers. What numbers were used for the actual citation counts?

 We also note that we find the suggestions by other reviewers compelling, and would be happy to review a revision of this manuscript should that be considered useful.

We have read this submission. We believe that we have an appropriate level of expertise to confirm that it is of an acceptable scientific standard, however we have significant reservations, as outlined above.

# Response

**Published:** 2016-09-30

**Authors:** Federico Vaggi

**Affiliations:** Fondazione Edmund Mach, Italy

We thank the reviewers for a very detailed examination of our manuscript.  They raise important issues, in particular, that certain findings which were exclusively correlations were treated as causative.  They also caught a minor mistake in the reported number of papers.

 We are currently in the process of submitting a revised manuscript that we believe addresses most of the issues they raise.

 For the generic comments:

 A)

 We tried to expand in more detail about what variables and models were used for the different analysis.  However, we believe that detailed descriptions are not as useful as mathematical formulas and the computer code that allows anyone to reproduce the analysis.  As a companion to the paper, we made a literate programming document (an IPython notebook) that shows and reproduces all the statistical analysis in the paper.

 B)

 We agree completely.  In the revised version of the paper, we tried to better explain the process through which an editor decides whether or not to serve as a reviewer.  Unfortunately, as this is a purely observational study without direct intervention, we cannot identify causal factors.

 We now address the specific questions:

 A)

 - As the reviewers correctly point out, the correct number of papers in the dataset is 2747, not 2750.  There were 3 other corrupted papers that were discarded that we accidentally included in the original count.  The papers that were dropped were those for which the database entries were corrupted (the data of resubmission was prior to the date where the original decision was made) or where data was missing.

 - For the citation file, we only added rows for papers that received citations (in scopus or otherwise).  When we merge the citation data with the other information about the paper, we implicitly treat all missing values as zeros (this can be seen in our script).  We did not imagine that people would try to manually reproduce the analysis using Excel - so we apologize if this caused additional difficulties.

 B)

 We downloaded all the different metrics that eLife makes available for all published papers (,Citations,Likes,en.search.wordpress.com,en.wikipedia.org,europepmc.org,f1000.com,scholar.google.com,twitter.com,www.citeulike.org,Pubmed_Citations,Scopus).  The column we used for all the analysis in the paper is Scopus.  We were surprised to find out that the different citation sources (Scopus, Pubmed, citeulike, etc) can have significantly different values.  This is important to take into account, as, unless discussed, this gives researchers a significant amount of degrees of freedom to pick the metric that best supports their hypothesis.

# Peer review: Referee response for version 1

**DOI:** 10.5256/f1000research.9100.r14223

**Published:** 2016-07-05

**Authors:** Bernd Pulverer

**Affiliations:** 1 European Molecular Biology Organization (EMBO), Heidelberg, Germany

Giordan *et al *analyzed 2,750 manuscripts sent out at the journal eLife for peer review (of which 1,405 ended up published). The authors compare papers in which the editors functions as ‘reviewing editor’ that is as one of three referees. Globally, and at almost every decision stage the process is accelerate significantly if the reviewing editor functions as one of the referees, with no or very small impact in author revision time and citation rates, respectively.

 The authors calculate that every additional external referee adds 7.4 days to the process and suggest that journals strive to balance the need for covering all the required expertises carefully with the negative effect on the speed of evaluation.

 The quality and speed of the peer review process are topics of active debate. Despite widespread criticism, the publication in certain peer reviewed journals continues to directly impact research assessment by both funders and institutions. The quality and fairness of the process is therefore paramount not only to assure the reliability of the literature, but also to inform research assessment in a balanced manner. Notwithstanding the slow delivery of this particular referee report, speed matters in particular in fast moving and highly competitive research areas like the biosciences.

 Quantitative evidence that well defined aspects of an editorial process has positive effect on quality and or speed is therefore of significant importance.

 The authors have carefully analyzed a decent sized dataset and report a statistically significant effect of a well defined change in the editorial process, while also showing evidence that this change has no detrimental effect on the quality of the editorial assessment, at least as far as the outcome is analyzed (here, in terms of two parameters: revision time and citation rate).

 While this manuscript makes a significant contribution, I have a number of suggestions I would invite the authors to consider in revision:

 Textural:

1. Abstract/main text; Background: It is not merely the growth of the number publications that puts the system under pressure (after all, in principle the editorial/peer review process may well be able to scale with increased research output), but rather the increased pressure publish in a small number of high Impact Factor journals in an effort to optimize chances of a positive impact on research assessment.
2. Please introduce the journal eLife, including the scientific scope, as different communities have widely different peer review and citation cultures and this will likely affect the findings reported here.
3. Abstract: Results. As presented, I found it confusing that the first sentence describes an apparently sizeable difference between accepted (10 days faster) and rejected (5 days faster), while the next sentence states ‘there was no effect on whether submissions were accepted or rejected)’.
4. Abstract: the dataset is described as consisting of an analysis of 8,905 submissions, when in reality the 2,750 papers sent for review were analyzed. This could be formulated more clearly.
5. I would suggest for clarity to remove the ‘False’ and ‘True’ nomenclature and change to ‘reviewing editor’ and ‘editor’ assessment or similar.

 

 Analysis:

1. It is unclear to me if the authors can exclude any biases in terms of which manuscripts were selected for formal review by an editor vs. outside only refereeing. Have the authors attempted to assess possible bias? For example, maybe the reviewing editors tend to review the manuscripts themselves that strike them as the more interesting ones, or maybe certain subject areas are preferentially subject to one approach.
2. I am missing a clear definition of what editorial peer review is and what is not. It is clear that this is likely not a completely binary situation and the authors do not describe how the decisions were parsed so clearly into the two groups (on p3. ‘more of a supervisory role’ vs. less of such a role sounds quite vague).
3. Why were appealed manuscripts removed form the analysis? This may introduce a bias as possibly erroneous decisions are excluded. How many appeals were excluded?
4. It is unclear to me if all paper invariably had three referees (i.e. 3 outside or 2 outside+reviewing ed.). I assume some papers only had two referees. Were they excluded? If not, how did these score for speed, revision time  and citation?
5. The definition of ‘Total Time’ on p3. Is unclear: it is stated to be both ‘first submission-acceptance’ and ‘first submission to publication’: which one is it?
6. Please state that Scopus citations were assessed when fist introducing the topic. I would recommend to use the same time window for all papers (e.g. 12 months after publication) as this renders citation rates more comparable. Why was the eLife website scraped for citation rates, and not the primary Scopus database – that data may in principle be more reliable.
7. The 10 days (accepted) vs. 5 days (rejected) faster: is this simply the additive effect of 2 rounds vs. 1 round of review?
8. Please include basic stats information in the figure legends – in particular fig 2, where the numbers will decrease dramatically for ‘revision 1’ and ‘revision 2’.
9. It is unclear to me if reviewing editors were invariably faster the outside referees. It would be useful to quantify this and assuming there is a striking difference to speculate why – it is the individuals selected by e-Life or due to policing or incentives provided by the journal? After all, similar strategies could be applied to outside referees. On a related point, it would be useful to quantify if the reports by the reviewing editors were qualitatively different (e.g. length). One assumes the ultimate decision on the manuscript was as also much better correlated with the reports by reviewing editors than those of the outside referees.
10. I am confused: in fig 2 ‘Reject full submission’ in revision 0 and revision 1 is slower than ‘accept’. This seems to be the opposite to fig 1 and in fact less intuitive than the results in fig 1.  Since manuscripts are rarely re-reviewed (see p1), are all the datapoints displayed in ‘revision 1’ and ‘revision 2’ for re-review processes?
11. For fig 4, I would suggest to plot the manuscript load/editor.

 

 Non-essential further reaching analysis (suggestions):

1. it would have been useful to measure and present the acceptance/rejection rates of manuscripts assess by three outside referees compared with two referees + reviewing editor.
2. it would have been useful to quantify the % of agreement between the reviewing editor and the outside referees, compared with agreement between the outside referees.

 

I have read this submission. I believe that I have an appropriate level of expertise to confirm that it is of an acceptable scientific standard, however I have significant reservations, as outlined above.

# Response

**Published:** 2016-09-30

**Authors:** Federico Vaggi

**Affiliations:** Fondazione Edmund Mach, Italy

We thank the reviewer for some very constructive comments, and for appreciating the manuscript.  We are now in the process of submitting a revised draft that we hope addresses all the major concerns.  Speaking as the corresponding author, I also completely agree that the current publishing system causes very major distortions in the behaviour of scientists who seek high impact publications for grants/tenure.  Unfortunately, given the currently economic climate and the funding situation for science, this is unlikely to change in the near future.

 By publishing this report, as well as making all the data available, we hope to at least make the process a bit more transparent, and give authors more information on how decisions are carried out.

 Textual Comments:

 1.  We agree completely, and emphasize this in the discussions.  

 2.  We have added more discussion in the text discussing eLife and its mission.

 3.  We tried to reword this sentence to make it more clear.

 4.  We rewrote this section.

 5.  We tried to have a more consistent nomenclature throughout the paper, including in the figures.

 Analysis Comments:

 1. The reviewer is completely correct on this, as other reviewers have also pointed out.  We tried addressing this in the text: the short answer is that while eLife tries to encourage all editors to act as reviewers on papers where they are qualified to do so, we cannot completely exclude that there is a selection process taking place.  We explain this in the text as well.

 2.  This was an unfortunate phrase, the situation is boolean.  Either the editor reviews the paper as a normal peer reviewer in addition to their duty as editor, or they purely play the role of editor.

 3.  Appeals were excluded because they were systematically corrupted in the database.  The dates for appealed papers were nearly all inconsistent - in the end, we removed them all because we couldn't be sure that even for those without inconsistent papers the dates were correct.

 4.  We discuss this at length in the text.  Some papers had only 2 reviewers.  They were not excluded from the analysis.  We examined the effect of the number of unique reviewers on citation rates as well as review time, including the papers with only 2 reviewers.

 5.  Fixed in the text.

 6.  We explained the use of Scopus more clearly.  We have also changed the figure showing citation rates more clearly: by using rates instead of total number of citations, we can normalize across time more easily.

 7.  Papers that were rejected had significantly fewer rounds of revision, so that's likely to be a significant cause of the shorter review time.  We also break down the data in detail in Figure 2, as well as Table 2.

 8.  As there is a lot of data condensed in those figures, we include a table in the text with all the numbers that are available.  Putting all the numbers inside the figure legend would make the text very hard to read.

 9.  This would be absolutely interesting to investigate further, but would require extensive resources to score individual reviews for quality.  We think this would be an excellent candidate for a follow up study, but falls slightly outside of the purpose of this work.

 10.  For the few paper that end up rejected after round 0, the review time is slightly longer for reject decisions than accept decisions.  Looking at Table 2 shows that the number of papers that make it to that stage is very small.

 11.  Great suggestion.  We have vastly changed Figure 4 to incorporate this feedback.

 Additional Suggestions:

 1.  This was analyzed in the linear model in Table S2.  We did not explicitly separate out the different categories, but treated each variable as continuous as we didn't find strong discontinuities in the data.

# Peer review: Referee response for version 1

**DOI:** 10.5256/f1000research.9100.r13794

**Published:** 2016-05-12

**Authors:** Alesia Zuccala

**Affiliations:** 1 Royal School of Library and Information Science, University of Copenhagen, Copenhagen, Denmark

I enjoyed reading this article, and I liked the fact that it was concisely written. I would only like to share a few comments.

The authors of this paper have chosen to exam the peer review process for *eLife*, which is an electronic journal for the life and biomedical sciences.  They note that two papers have previously described the editorial process of *eLife*; however for their own study, I think it would have been useful to include a link to the *eLife* website ( https://elifesciences.org/).   I was curious about whether or not it was an open access journal, and I looked for the website to obtain this information.   More importantly, I also looked to the website, because I wanted to know how many persons serve as Senior Editors or sit on the editorial board of *eLife*.  What is interesting here is that the term “editor” for this journal is stretched to include one Editor-in-Chief, three Deputy-Editors, thirty-two Senior Editors and a 282-member Board of Reviewing Editors.   The peer review system for this journal is quite different from the ‘traditional’ journal, but to be more precise, it differs because “Reviewing Editors” are specialists who have agreed to review for the journal on a regular basis, and may in some cases call upon additional ‘outside’ reviewers. 

What we do not know from this paper, is whether or not two or more of the 282 Reviewing Editors sometimes choose to review the same paper.  At the *eLife* website, the following is noted:  “The Reviewing editor usually reviews the article him or herself, calling on one or two additional reviewers as needed”.  Are the additional reviewers always from the outside?  If not, how would this change the authors’ hypothesis related to the ‘effects of an editor serving as one of the reviewers’?

The methods used for the data analysis are explained very well, with the exception of one detail:  How did the authors acquire the initial dataset of 9,589 papers?   This information is presented in the ‘Acknowledgements’ section, but could have also been added to the Methods section, for more clarity.

The graphs related to the authors’ findings are clear and present interesting information, but I am not sure how the citation data were collected from Scopus for the peer-reviewed papers in *eLife *and whether or not ‘citation windows’ were used for the papers depending on the year in which they were published.  Essentially the authors are correct in saying that “ *papers accumulate citations over time, and, as such, papers published earlier tend to have more citations*”, hence citation windows are used to correct for this.  The highest rates of citation (especially in the life sciences and biomedicine) will appear within three-to-five years following an article’s date of publication.  For this reason, bibliometricians usually count citations within this three-to-five year time-frame to determine an article’s initial impact.   Since the articles used in this study had been “ *submitted to eLife since June 2012*” the authors should have focus on three things:  1) the involvement of a Reviewing Editor as a peer reviewer or not, 2) the number of days between start of the submitted paper’s acceptance and publication, and 3) the papers’ citation rate following 3-5 years after final publication.

I have read this submission. I believe that I have an appropriate level of expertise to confirm that it is of an acceptable scientific standard.
